# Anti-Onchocercal Properties of Extracts of *Scoparia dulcis* and *Cylicodiscus gabunensis*

**DOI:** 10.1155/2022/4279689

**Published:** 2022-11-16

**Authors:** Tiku Edward Tiku, Moses Samje, Napoleon Mfonku, Fidelis Cho-Ngwa

**Affiliations:** ^1^Department of Biochemistry and Molecular Biology, University of Buea, P. O Box 63 Buea, South West Region, Buea, Cameroon; ^2^Department of Biomedical Sciences, University of Bamenda, Bamenda, Cameroon; ^3^Department of Chemistry, University of Buea, S. W Region, Buea, Cameroon

## Abstract

**Introduction:**

The elimination of onchocerciasis is hampered by the absence of suitable drugs that are effective against adult filariae. This study is aimed at assessing the anti-onchocercal effects of extracts of *Scoparia dulcis* and *Cylicodiscus gabunensis* that could serve as drug leads against onchocerciasis.

**Methods:**

Different parts of the plants (*Scoparia dulcis* and *Cylicodiscus gabunensis*) were extracted with hexane, methylene chloride, and methanol. The extracts were tested *in vitro* against the bovine model parasite, *Onchocerca ochengi*. Adult female worm viability was determined biochemically by MTT/formazan colorimetry, while the adult male and microfilariae viability were determined by microscopy based on % inhibition of worm motility score. Cytotoxicity and acute toxicity of active extracts were tested on monkey kidney epithelial cells (LLC-MK2) and Balb/C mice, respectively.

**Results:**

The hexane extract of *Scoparia dulcis* recorded the highest activity, with IC_50s_ of 50.78 *μ*g/ml on both adult male and female worms and 3.91 *μ*g/ml on microfilariae. For *Cylicodiscus gabunensis* extract, the highest activity was seen with the methylene chloride extract, with IC_50s_ of 50.78 *μ*g/ml, 62.50 *μ*g/ml, and 16.28 *μ*g/ml on, respectively, adult male, female, and microfilariae. The 50% cytotoxic concentration on the LLC-MK2 cells was 31.25 *μ*g/ml for the most active extracts. No acute toxicity was recorded for the extracts. Phytochemical analysis of the extracts revealed the presence of alkaloids, flavonoids, sterols, saponins, phenols, and glycosides.

**Conclusion:**

This study validates the traditional use of these plants in treating onchocerciasis and suggests *S. dulcis* and *C. gabunensis* as new potential sources for the isolation of anti-onchocerca lead compounds.

## 1. Introduction

Medicinal plants are widely used in traditional cultures all over the world and are becoming increasingly popular in modern society to meet health care needs, particularly in Africa and most developing countries [1]. Natural product molecules represented more than 50% of drugs that had been put into the drug market, with many medicinal plants extracts now used as prescription drugs in many developed countries [2–4]. Approximately, a quarter of all Food and Drug Administration and European Medical Agency (EMA) approved drugs are plant-based, with well-known drugs such as quinine, artemisinin, morphine, and the anticancer drugs paclitaxel and vincristine [5–8]. The presence of phytoconstituents gives plant species their medicinal potential with numerous functions such as antimicrobial, anticancer, antioxidant, and antiviral [1, 9].

*Scoparia dulcis* is a herb of the family Plantaginaceae that is common in Tropical Africa, Asia, and Central America. It is used traditionally to treat fever, cold, sore throat, and eczema, with other ethnomedical uses like the treatment of gastric problems, reproductive issues, piles, liver, and respiratory diseases [10, 11]. Approximately, 160 compounds have been identified from *S. dulcis*, among which 155 have been related to the treatment of metabolic syndromes [10]. Previous chemical analysis of the plant has identified various phytoconstituents such as nitrogen-containing compounds, flavonoids, diterpenoids, triterpenoids, steroids, phenolics, and aliphatics, with several pharmacological effects such as been antidiabetics, anticancer, antiarthritic, antihyperlipidaemia, anti-inflammatory, and antiurolithiasis [10, 12, 13].

*Cylicodiscus gabunensis* (Fabaceae) is an indigenous medicinal plant that is widely distributed in West and Central Africa. Extracts from its stem bark have been used for the treatment of viginitis, jaundice, antimalaria, antibacteria, anti-inflammatory, antipyretics, and as soaps and mouthwash by some communities [14–17]. Previous phytochemical analysis has revealed the presence of triterpenoids, saponins, and phenolics with some definite pharmacological antimalaria, antifree radical, and antibacteria properties [16, 18, 19]. Ethnopharmacological information about *S. dulcis* and *C. gabunensis* indicated their use by an ethnic group in Cameroon to treat filarial infections like onchocerciasis.

Human onchocerciasis, or river blindness, is a neglected tropical disease with serious debilitating effects, affecting over 37 million people worldwide, mostly in developing countries, including Yemen and many countries found in Africa, Central and South America [20]. Over 200 million people are at risk of onchocerciasis infection, and more than 99% of the disease burden is found in Africa, with an estimated 1.15 million of the infected being blind and an additional 500, 000 living with severe visual impairment [21–23]. The disease is caused by the filarial parasitic nematode *Onchocerca volvulus*, with the Simulium blackfly being its vector. Despite several efforts to control the disease, limitations are found in new strategies for blocking transmission [24]. Ivermectin remains the only drug currently recommended for the treatment and control of the disease. The drug mainly targets the microfilarial (juvenile) stage of the parasite, leaving the adult worms to continue to reproduce. There is also a serious adverse effect in individuals who are treated with ivermectin and are coinfected with *Loa loa* with high microfilaraemia (greater than 30 000 microfilariae per ml) [25]. Reports on the resistance of the parasites to some of the filaricidal drugs, coupled with some of their drawbacks and restrictions of use in some coendemic areas, have led to the urgent search for alternative treatments with macrofilaricidal properties [7, 25]. One strategy employed has been the exploitation of medicinal plants for the identification of novel potential drug leads. This study investigated the filaricidal properties of *Scoparia dulcis* (Plantaginaceae) and *Cylicodiscus gabunensis* (Fabaceae) on cattle derived from *Onchocerca ochengi* (the closest known relative of *Onchocerca volvulus*), and their possible use as sources of new drug leads for the treatment of onchocerciasis.

## 2. Materials and Methods

### 2.1. Ethics Approval and Consent to Participate

Ethical clearance (2019/018/UB/IACUC/BTU/FS) for the use of the animals was obtained from the Institutional Animal Care and Use Committee (UB-IACUC), University of Buea, Cameroon.

### 2.2. Collection and Identification of Plant Materials

Aerial parts of *Scoparia dulcis* and stem bark of *Cylicodiscus gabunensis* were collected from Okpambe village in the Takamanda area in the Manyu Division South West Region of Cameroon in March 2016. The plants were selected based on ethnopharmacological information about them. They were identified and authenticated at the Limbe Botanic Garden, South West Region of Cameroon, and given the voucher numbers, SCA 1225 and SCA 5092, for *Scoparia dulcis* and *Cylicodiscus gabunensis*, respectively.

### 2.3. Preparation of Plant Crude Extracts

The harvested plants were air-dried and ground to a fine powder. The ground material was weighed and subsequently immersed and macerated for 72 hours in three different solvents, namely: hexane (Hex), methylene chloride (MeC), and methanol (MeOH). For each solvent, the maceration was repeated twice. The mixture was filtered and the filtrate was concentrated using a rotary evaporator (BUCHI Rotavapor R-200, Switzerland) at appropriate temperatures. The concentrates were recovered with methylene chloride and allowed to stand at room temperature until the residual solvents had evaporated. The dried crude extracts were stored at −20°C until needed for assays. Stock solutions of 25 mg/ml of the different plant extracts were prepared in >99.8% dimethyl sulfoxide (DMSO) (Sigma, USA) and stored at −20°C until tested in biological assays.

### 2.4. Screening against *O. ochengi* Adult Worms

The adult worms were extracted according to Cho-Ngwa et al. 2010. Briefly, subcutaneous nodules containing adult *O. ochengi* worms were identified on the umbilical skin of infected cows and immediately brought to the laboratory. Under sterile conditions, adult worm masses containing one viable adult female and zero to several adult males were carefully recovered by dissection of the nodule with a sterile razor blade. The masses were then incubated in 2 ml of complete culture medium (CCM), which comprised RPMI-1640 (Sigma-Aldrich, U.S.A), 10% newborn calf serum, 200 units/ml penicillin, 200 *μ*g/ml streptomycin, and 2.5 *μ*g/ml amphotericin B (Sigma-Aldrich, U.S.A) in standard 12-well culture plates (NUNC, USA) and incubated overnight at 37°C in a 5% CO_2_ incubator (Thermo Fisher, UK). The next day, 1000 *μ*g/ml of the different plant extracts was prepared in 2 ml of CCM to generate a final of 500 *μ*g/ml for the primary screening. The extracts and controls were tested in triplicate at each concentration, and the experiment was repeated trice on different days. The negative control wells contained only the diluent, DMSO (500 *μ*g/ml). Cultures were terminated on day 7, post addition of plant extracts. Adult male worm viability was visually scored using an inverted microscope (Nikon Eclipse TS200, China) on days 5 and 7, with a percentage reduction of motility ranging from 100% (complete inhibition of motility), 75% (only head or tail of worm motility), 50% (whole body of worm motile but sluggishly), 25% (almost vigorous motility) to 0% (no observable reduction in motility) [26]. Adult female worm viability was assessed on day 7 by the standard MTT/Formazan assay in which each nodular mass was placed in a well of a 48-well microtitre plate containing 500 *μ*L of 0.5 mg/ml MTT (Sigma-Aldrich, U.S.A) in incomplete culture medium (ICM) (composed of RPMI-1640 (Sigma-Aldrich, U.S.A), 200units/ml penicillin, 200 *μ*g/ml streptomycin, and 2.5 *μ*g/ml amphotericin B, and then incubated in the dark at 37°C for 30 minutes. Adult female worm viability was evaluated visually by the extent to which the female worm mass was stained with MTT. Mean percent inhibition of formazan formation was calculated relative to the negative control worm mass. Adult worm death is positively correlated with the inhibition of formazan formation. The positive control was auranofin at 30 *μ*M [25]. Secondary screening for the active extracts (100% activity) was done to determine the IC_50_ for the extracts. The active extracts were retested as described under the primary screen at serial dilutions of seven concentrations (from 500 to 7.8125 *μ*g/ml). Assays were done in triplicate and each experiment was repeated for confirmation. The means of all activities at a concentration were calculated using the statistical analysis Graphpad Prism version 6.0 (Graphpad Software, CA, USA) to generate dose-response curves from which the IC_50_ values were obtained.

### 2.5. Screening against *O. ochengi* Microfilariae *in Vitro*

The mammalian kidney cells (LLC-MK2) obtained from the American Type Culture Collection (ATCC, Virginia, USA) were proliferated in CCM at 37°C under an atmosphere of 5% CO_2_ in humidified air. The cells were seeded in 96-well plates until they became fully confluent and served as feeder layers for the mf assays. The cells were also used for cytotoxicity assessment of the extract [27]. *O. ochengi* microfilariae were isolated by the method of Cho-Ngwa et al. [28] with slight modifications. Briefly, umbilical cattle skin pieces containing palpable nodules were obtained from the abattoir, cleaned, carefully shaved, and sterilized with 70% ethanol. Skin slivers were obtained and incubated for 4–6 hours at room temperature in CCM. The emerged *O. ochengi* mf was concentrated by centrifugation (400xg, 10 min.). The highly motile mf was resuspended in CCM and distributed into wells (approximately 15mf/100 *μ*l of CCM/well) of 96-well plates containing the LLC-MK2 cell layer, and their viability and sterility were ascertained at the 24th hour prior to the addition of extracts.

The primary screens for *O. ochengi* mf were done at 500 *μ*g/ml in duplicates to eliminate inactive extracts. Extracts that showed 100% activity were retested in the secondary screening as described above for the adult worm assay to determine the IC_50_ values. The mf was incubated at 37°C under an atmosphere of 5% CO_2_ in humidified air for 5 days. The positive control was amocarzine (5 mM), while the negative control contained 2% DMSO. Mf motility was scored microscopically daily. The percentage motility inhibition scores were assigned as 100% (all mf immotile), 75% (only head or tail of mf shaking occasionally), 50% (whole body of mf motile but sluggishly or with difficulty), 25% (almost vigorous motility), and 0% (vigorous motility) [28, 29].

### 2.6. Cytotoxicity Studies

This experiment was conducted on the active extracts only. The fully confluent cells were cultured in the presence of the extracts at varying concentrations and observed daily under an inverted microscope. By day 7, an MTT colorimetric assay was done on the cells [30]. Succinctly, MTT colorimetric assay involved the culturing of LLC-MK2 cells in CCM in a 96-well flat bottom plate, until when the cells attain a density of approximately, 3000 cells/100 *μ*l of CCM. Thereafter, the culture media of the fully confluent cells was removed by inverting, flicking, and blotting the plate, followed by the addition of crude extracts in fresh CCM. Colored extracts were washed twice with 100 *μ*L of ICM. A 5 mg/ml MTT stock in 1x phosphate buffer saline was prepared and diluted in ICM to obtain a concentration of 1 mg/ml. To the cells in each well was added 100 *μ*l of the 1 mg/ml solution of MTT. The plate was incubated for 3 hours at 37°C in a humidified 5% CO_2_ incubator. At the end of incubation, the MTT solution was removed by inverted flicking and blotting of the plate. One hundred microliters (100 *μ*l) of DMSO was added to each well and the plate was shaken at 400 rpm for 5 minutes. Inhibition of formazan formation with MTT directly correlates with cell death, purple formazan formation indicated viable cells, while pale purple correlated with the IC_50_ of the cells [30].

Under an inverted microscope (Nikon Eclipse TS200, China), living cells are flattened out and attached to the culture plate, while dead cells are rounded up and detached from the bottom of the plate.

The selectivity index (SI) values were calculated using the ratio as follows:

(1) $$\begin{array}{c} \text{SI}=\frac{{\text{CC}}_{50}\text{ of mammalian cells}}{{\text{IC}}_{50}\text{of parasite}}. \end{array}$$

### 2.7. Acute Toxicity Studies

The test was conducted in accordance with the OECD guidelines for testing of chemicals [31] and the animal protocol was approved by The Animal Care and Use Committee, of the Faculty of Science, University of Buea. The extracts with antifilaricidal activity were tested for acute toxicity in BALB/*c* mice. A total of 12 animals of approximately 20 g of body weight each were used and divided into 2 groups, 6 for each extract and of equal sex. Each of the 2 treatment groups received one of the extracts at a limited dose of 2000 mg/kg body weight, administered orally in a maximum volume of 250 *μ*l of vegetable oil per animal, while the control group received the diluent only. The animals were observed daily for 14 days for any changes in physical activity, food intake, and water intake, loss of fur, stool samples, sensitivity to sound, and sensitivity to pain, motility, and mortality.

### 2.8. Phytochemical Analysis

The phytochemical properties of the active extracts were determined qualitatively using standard procedures: frothing test for saponins, Fehling's test for glycosides, Meyer's and Dragendoff's reagents for alkaloids, acetic anhydride and sulphuric acid reagents for steroids, ferric chloride test for tannins, aluminium chloride test for flavinoids, and ferric chloride and alcohol test for phenols [32].

### 2.9. Statistical Analysis

Data were analyzed using Graphpad Prism version 6 (Graphpad Software, CA, USA) to determine the IC_50s_ and mean motility scores.

## 3. Results and Discussion

**Figure 1:**
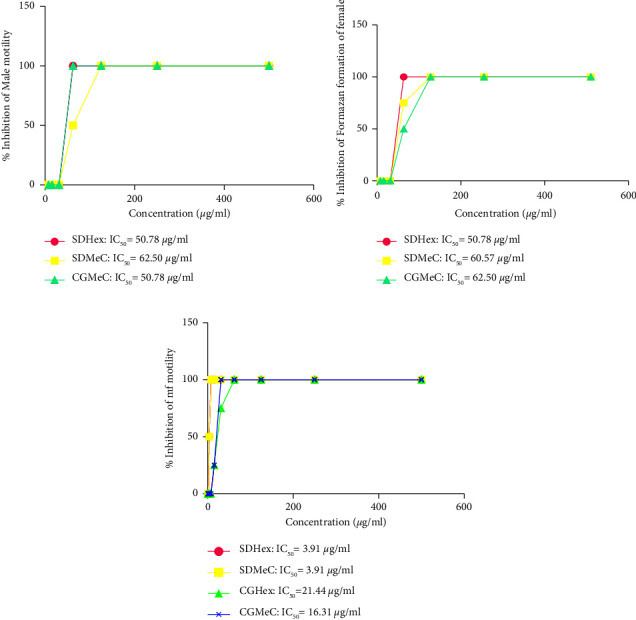
(a) % inhibition of *O*. *ochengi* adult male motility for SDHex, SDMec and CGMeC (b) % inhibition of formazan formation on *O*. *ochengi* adult female motility for SDHex, SDMec and CGMeC (c) % inhibition of *O*. *ochengi* mf motility.

A total of 6 crude extracts were prepared from the two plants using solvents of different polarities (hexane, methylene chloride, and methanol). Results of the primary screen showed that the hexane and methylene chloride extracts of the two plants were more effective in killing the adult *Onchocerca ochengi* male, female, and microfilaria with IC_50_ values within the range of 62.5–3.91 *μ*g/ml (Table 1). The hexane extract of *Scoparia dulcis* was more active against adult male and female worms, with an IC_50_ value of 50.78 *μ*g/ml, while the methylene chloride extract was active with IC_50_ values of 62.50 *μ*g/ml and 60.57 *μ*g/ml on male and female *O. ochengi*, respectively. Both extracts exhibited the same activity on microfilariae, with an IC_50_ value of 3.91 *μ*g/ml (Figures 1(a)–1(c)). The methylene chloride extracts of *Cylicodiscus gabunensis* were active against adult male, female, and microfilariae of *O. ochengi* with IC_50_ values of 50.78 *μ*g/ml, 62.50 *μ*g/ml and 16.28 *μ*g/ml, respectively, while the hexane extract recorded an IC_50_ value of 22.10 *μ*g/ml for the *O. ochengi* microfilaria (Figures 1(a)–1(c)).

**Figure 2:**
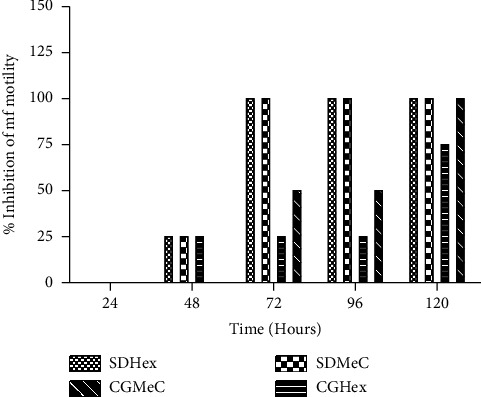
Time-dependent inhibition of *O*. *ochengi* mf motility at 7.81 *μ*g/ml and 31.25 *μ*g/ml for SDHex; SDMeC and CGHex; CGMeC respectively.

The time-dependent studies of SDHex and SDMeC demonstrated a 100% inhibition of mf motility on the 72^nd^ hour for both extracts at 7.81 *μ*g/ml, while those of CGHex and CGMeC showed 75% and 100% inhibition, respectively, for mf motility on the 120^th^ hour at 31.25 *μ*g/ml (Figure 2).

### 3.1. Cytotoxicity and Acute Toxicity of Active Extracts

The drug concentration inducing cytotoxicity in 50% of cells (CC_50_) was 31.25 *μ*g/ml for both extracts of *S. dulcis*, while the CC_50_ for *C. gabunensis* was 3.0 *μ*g/ml and 6.5 *μ*g/ml for hexane and methylene chloride extracts, respectively. The Selectivity Index (SI) values of the extracts for adult worms and mf of the parasite ranged from 0.1 to 7.99 (Table 2). In general, the cytotoxicity assay demonstrated that for *S. dulcis* extracts, the adult worms had SI values below 1, while the mf had SI values above 1. The SI values for the *C. gabunensis* extracts were all below 1.

The active extracts were selected for acute toxicity studies in BALB/C mice at a limited dose of 2000 mg/kg of body weight. Following the administration of the test substance to that of the control mice, no change was observed in the physical activity and behavior of the test and control animals. The test and control groups were indistinguishable from one another on the basis of their appearance and physical activity at the end of the 14 days study period.

### 3.2. Phytochemical Composition of Active Extracts

Phytochemical screening revealed the different classes of secondary metabolites present in the active hexane and methylene chloride extracts of *S. dulcis* and *C. gabunensis*. The *S. dulcis* extracts showed positive for the presence of steroids, glycosides, phenolics, and flavonoids (Table 3), with alkaloids and saponins present only in the hexane and methylene chloride extracts, respectively. The *C. gabunensis* extracts showed positive for the presence of steroids, glycosides, phenolics, and flavinoids (Table 3), with alkaloids and saponins present only in the hexane and methylene chloride extracts, respectively.

## 4. Discussion

The aim of this study was to appraise the anti-onchocercal activity of two medicinal plants–*Scoparia dulcis* and *Cylicodiscus gabunensis*. From the ethnopharmacological survey, the plants were selected for the study based on their traditional claim to manage filariasis by traditional health practitioners in Okpambe; a village in the Takamanda area in the Manyu Division of the South West Region Cameroon. There has not been any scientific report on the use of these plants and its metabolites for the management of filaria infections. Previous studies of the plants have been reported for their use as antidiabetes, anti-inflammatory, antimicrobial, antisickling, antioxidant, antimalarial, and in the treatment of skin diseases, warts, jaundice, dysentery, gonorrhea, snake bites, and gastrointestinal disorders [11, 15, 33, 34]. *Onchocerca ochengi* was considered the most suitable model for screening anti-onchocerca phytomedicines because it is the closest relative to *Onchocerca volvulus* in which both parasite shares the same Simulium vector, in addition to the low cost of obtaining *O. ochengi* and the ease of its availability [25, 26].

The primary screen was done *in vitro* on all stages of the *Onchocerca ochengi* parasite for the hexane, methylene chloride, and methanol extracts of *S. dulcis* and *C. gabunensis* plants. The hexane and methylene chloride extracts showed 100% activity at 500 *μ*g/ml against the stages of the parasite. The extracts were subjected to a secondary screen at lower doses, ranging from 500 *μ*g/ml down to 7.8125 *μ*g/ml. This gave a promising IC_50_ values within the range 62.5–3.91 *μ*g/ml. The hexane extract of *S. dulcis* was active against the adult male and female *O. ochengi* with IC_50_ value of 50.78 *μ*g/ml, while the methylene chloride extract showed activity with IC_50_ values of 62.50 *μ*g/ml and 60. 57 *μ*g/ml on the male and female *O. ochengi*, respectively. The methylene chloride extract of *C. gabunensis* was active against the adult male and female of the parasite, with IC_50_ values of 50.78 *μ*g/ml and 62.50 *μ*g/ml respectively, (Figures 1(a) and 1(b)). The activity against the adult onchocerca worms is an important finding because as of date, the lone approved recommended drug for the treatment of the disease does not kill the adult worms (macrofilariae), leaving it to continue to reproduce and produces the microfilariae (juvenile worms) that generate pathologies [25]. The hexane and methylene chloride extracts of *S. dulcis* exhibited the same activity on the microfilariae with an IC_50_ value of 3.91 *μ*g/ml, while the hexane extract of *C. gabunensis* recorded an IC_50_ value of 22.10 *μ*g/ml (Figure 1(c)). These results add information in support of previous findings that showed nonpolar extracts to be nematocidal more than polar ones [27, 35, 36]. The seemingly same IC_50_ values of both extracts on the male and female Onchocerca parasites might be due to the fact that both parasites have the same targets for the active plant extracts, while the discrepancy in activity between the adult and juvenile parasites probably indicates a variation in the target of the different extracts. The juvenile form of the parasites was more liable to be killed than the adult worms. This might be due to the fact that the juvenile worms are not entangled in nodular masses like adult worms, giving them more exposure to the compounds than adult parasites [29]. In addition, the fact that the extracts could kill the different parasitic stages of *O. ochengi* making these plants a potential source for the isolation of novel compounds with anti-onchocerca activity.

The safety of the plant extracts was done by evaluating cytotoxicity in LLC-MK2 cells and acute toxicity in Balb/*c* mice. The selectivity index of *S. dulcis* extracts was less than 1 for the adult parasites and 7.99 (greater than 1) for the mf, while that of the *C. gabunensis* extracts was all less than 1. These values indicate that the hexane and methylene chloride extracts of *S. dulcis* and the extracts of *C. gabunensis* were more toxic to the LLC-MK2 cells with respect to the adult parasites, while the hexane and methylene chloride extracts of *S. dulcis* were more toxic to the juvenile parasites than the LLC-MK2 cells. The high toxicity of the extracts on the adult parasites might be due to the complex morphology of the adult parasites relative to the LLC-MK2 cells, where a fairly high concentration of active molecules is required for feasible responses in activity. We observe that none of the laboratory mice died after administration of the active extracts at a limited dose during acute toxicity studies. This finding corroborates previous toxicology studies of these plant extracts, which demonstrated no toxicity to laboratory rats [11]. The use of these plants in the treatment of filarial and other infections by the indigenes of Okpambe and other indigenous populations over centuries without serious side effects is proof of their safety. The absence of adverse effects in patients who take the plant concoctions for therapy might be due to *in vivo* detoxification mechanisms, which are absent in *in vitro* studies.

Phytochemical analysis of *S. dulcis* and *C. gabunensis* revealed the presence of secondary metabolites; flavonoids, sterols, phenols, and glycosides in both extracts of the plants, and alkaloids in the hexane extracts and saponins in the methylene chloride extracts. This suggests that the active principles in the extracts may be from the groups of compounds mentioned above. This is in conformity with previous phytochemical studies, which revealed the presence of these secondary metabolites to be responsible for the medicinal properties of these plants [16, 33].

The isolation of pure compounds from these plant extracts is recommended to determine their full active potential and ascertain the actual phytochemicals responsible for the anti-onchocerca activity of the plant extracts. Such refinement might enhance the activity of the lead compounds against the parasite, and possible reaction mechanisms could be elucidated from any pure lead compound isolated with an attribute of anti-onchocerca activities.

Remarkably, none of the *S. dulcis* and *C. gabunensis* extracts have been previously tested against Onchocerca and other filaria species. Our data reinforce the existing knowledge and regular use of these plants by traditional health practitioners for the treatment of infectious diseases.

## 5. Conclusions

This study has revealed for the first time the anti-onchocercal activity of extracts of *S. dulcis* and *C. gabunensis*, which could serve as potential novel sources of new drugs against onchocerciasis. It also validates the use of these plants by traditional health practitioners in the local management of filariasis.

## Figures and Tables

**Table 1:** IC_50_ and IC_100_ values of extracts in the secondary screen for male, female, and microfilariae (mf) of *Onchocerca ochengi*.

Extracts Code	IC_50_ (*μ*g/ml) for % inhibition of male worm motility	IC_100_ (*μ*g/ml) for % inhibition of male worm motility	IC_50_ (*μ*g/ml) for % inhibition of female worm	IC_100_ (*μ*g/ml) for % inhibition of female worm	IC_50_ (*μ*g/ml) for % inhibition of microfilariae motility	IC_100_ (*μ*g/ml) for % inhibition of microfilariae motility
SDHex	50.78	62.50	50.78	62.50	3.91	7.81
SDMeC	62.5	125	60.57	125	3.91	7.81
CGMeC	50.78	62.5	62.5	125	16.28	31.25
CGHex	—	—	—	—	22.10	62.50

(SDHex: hexane extract of *S. dulcis*; SDMeC: methylene chloride extract of *S. dulcis*; CGMeC: methylene chloride extract of *C. gabunensis*; CGHex: hexane extract of *C. gabunensis*).

**Table 2:** Selectivity index (SI) values of extracts of *S. dulcis* and *C. gabunensis* against the parasite stages.

*O. ochengi Parasite* stages	SDHex CC_50_: 31.25 *μ*g/ml IC_50_ (*μ*g/ml) S.I		SDMeC CC_50_: 31.25 *μ*g/ml IC_50_ (*μ*g/ml) S.I		CGHEX CC_50_: 3.0 *μ*g/ml IC_50_ (*μ*g/ml) S.I		CGMeC CC_50_: 6.5 *μ*g/ml IC_50_ (*μ*g/ml) S.I	
Male	50.78	0.62	62.5	0.50	—	—	50.78	0.13
Female	50.78	0.62	60.57	0.52	—	—	62.5	0.1
Microfilariae	3.91	7.99	3.91	7.99	22.1	0.14	16.28	0.4

**Table 3:** Phytochemical analysis of hexane and methylene chloride extracts of *S. dulcis* and *C. gabunensis.*

Extracts	Alkaloids	Flavonoids	Sterols	Saponins	Phenols	Glycosides	Tannins
SDHex	+	+	+++	−	++	++	−
SDMeC	−	++	++	+	+	+++	−
CGHex	+++	++	+++	−	+	−	−
CGMeC	−	++	+++	+	++	+	−

−: Absent, +: Low, ++: Moderate, +++: Abundant.

## Data Availability

The data used to support the findings of this study are included within the article.
